# Arsenic Trioxide exerts cytotoxic and radiosensitizing effects in pediatric Medulloblastoma cell lines of SHH Subgroup

**DOI:** 10.1038/s41598-020-63808-9

**Published:** 2020-04-22

**Authors:** Paulo Henrique dos Santos Klinger, Lara Elis Alberici Delsin, Gustavo Alencastro Veiga Cruzeiro, Augusto Faria Andrade, Regia Caroline Peixoto Lira, Pamela Viani de Andrade, Pablo Ferreira das Chagas, Rosane Gomes de Paula Queiroz, Felipe Amstalden Trevisan, Ricardo Santos de Oliveira, Carlos Alberto Scrideli, Luiz Gonzaga Tone, Elvis Terci Valera

**Affiliations:** 1 Department of Pediatrics, University of São Paulo, Ribeirão Preto, Brazil; 2 Department of Genetics, University of São Paulo, Ribeirão Preto, Brazil; 3 Division of Radiotherapy, University of São Paulo, Ribeirão Preto, Brazil; 4 Division of Pediatric Neurosurgery, Department of Surgery and Anatomy; Faculty of Medicine of Ribeirão Preto, University of São Paulo, Ribeirão Preto, Brazil; 5 Instituto de Oncologia Pediátrica IOP/GRAACC, São Paulo, Brazil; 6 Centro Universitário CESMAC, Maceió-AL, Brazil

**Keywords:** Apoptosis, CNS cancer

## Abstract

We evaluated the potential effects of ATO in different pediatric SHH-MB cell lines (ONS-76: *TP53*-wild type; DAOY and UW402: *TP53*-mutated). MB cell lines molecular subgroup was confirmed and *TP53* mutations were validated. Cell viability, clonogenicity and apoptosis were evaluated after ATO treatment at different concentrations (1–16 µM) alone or combined with irradiation doses (0.5, 1, 2 and 4 Gy). Rad51 and Ku86 proteins were evaluated by WB. ATO treatment reduced cell viability for all SHH-MB cell lines. Significant decrease of clonogenic capacity and higher apoptosis rates were also observed after ATO exposure, being cell death more pronounced (>70%) for the SHH-MB *TP53*-mutated. Combined treatment of ATO with irradiation also reduced colonies formation in UW402 tumor cells, which was independent of DNA damage repair proteins Rad51 and Ku86. In silico analyses suggested that a set of genes from cell cycle and p53 pathways are differentially expressed in SHH tumor subtypes, suggesting that cell lines may respond to therapies according to the gene expression profiles. Herein, we showed ATO cytotoxicity in pediatric SHH cell lines, with marked radiosensitizing effect for the MB-SHH *TP53*-mutated cells. These results highlight the potential of ATO, alone or in combination with radiotherapy, supporting further clinical investigations.

## Introduction

Medulloblastoma (MB) is the most common malignant brain tumor in children, corresponding to approximately 20% of all brain tumors in patients less than 15 years of age. MB is responsible for significant morbidity and mortality rates^1^. Recently, twelve distinct subgroups allocated to four main molecular classes (SHH, WNT, Group 3 and Group 4) have been recognized for MB: WNT-alpha and beta; SHH-alpha, beta, gamma and delta; Group 3-alpha, beta and gamma; and Group 4-alpha, beta, and gamma^2^. Considering the clinical significance of the MB molecular classification, new therapies that contemplate the genetic aspects of the disease are required^3^.

The majority of SHH-MB cases presents somatic mutation in one or more genes of the Sonic Hedgehog (SHH) pathway (i.e., *PTCH1*, *SUFU*, or *SMO*), contributing to its constitutive activation. SHH signaling is essential for embryonic development, as well as it can lead to a tumor arising when aberrantly activated^4^. The SHH-MB subgroup prognosis in children is mostly favorable, but the clinical outcome varies within this subgroup. SHH-MB alpha cases are rarely observed in infants and carry the poorest prognosis, which is associated with *MYCN* and *GLI* (glioma-associated oncogene homolog) amplification, and higher *TP53* mutational burden. In contrast, SHH-MB beta and gamma subtypes affect mainly infants, being one-third of the SHH-MB beta cases metastatic at presentation with frequent focal PTEN deletions. The SHH-MB gamma is strongly related to MB with extensive nodularity (MBEN) histology, in general, presents wild type *TP53*, and excellent prognosis^2^. Lastly, the SHH-MB delta is frequently seen in adults, has a favorable outcome, and is recurrently enriched in *TERT* promoter mutations^2,5^.

New targeted-therapies strategies for the poor prognostic subgroups of MB are necessary. The Arsenic trioxide (ATO) is a well-known drug with therapeutic effects on acute promyelocytic leukemia (APL). The binding, oxidation and sumoylation of ATO on PML nuclear bodies or the RNF4-mediated ubiquitination contribute to the catabolism of the APL oncoprotein PML/RARA^6^. ATO also induces the generation of reactive oxygen species, inducing apoptosis and cell cycle arrest^6^. Although ATO has a well-established effect over SHH pathway and reasonable oral absorption with good penetration in the central nervous system (CNS)^7,8^ its role as SHH-MB targeted therapy, alone or in combination with irradiation, has not been reported to date^9,10^.

## Results

### ATO controls cell viability, induces apoptosis and improves radiosensitivity in SHH-MB cells

**Figure 1:**
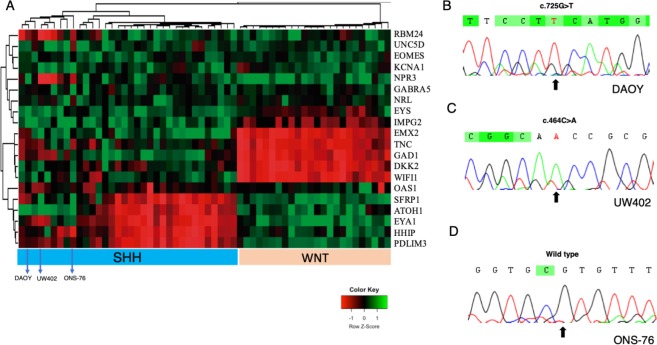
(**A**) Hierarchical unsupervised clustering of cell lines DAOY, UW402 and ONS-76 along with medulloblastoma samples assigned as SHH (blue) and WNT (pink) subgroup. Pearson distance followed by average-linkage algorithm was utilized as clustering parameters. (This figure was modified from the original version in Cruzeiro *et al*., 2019); **(B)** Eletropherogram of *TP53* mutation loci in DAOY cell line (c.725G > T); **(C)** Eletropherogram of *TP53* mutation loci in UW402 cell line (c.464C > A) **(D)** Eletropherogram of *TP53* Wild-type loci in ONS-76 cell line.

The MB molecular profile of the three MB cell lines models (DAOY, UW402 and ONS-76) was validated by TDLA, which confirmed the SHH molecular subgroup (Fig. 1A). Regarding the *TP53* status, Sanger sequencing confirmed mutations in DAOY (*TP53* - c.725G > T) and UW402 (*TP53* - c.464C > A), while the ONS-76 cell line was shown to be SHH *TP53* wild type (Fig. 1B–D).

**Figure 2:**
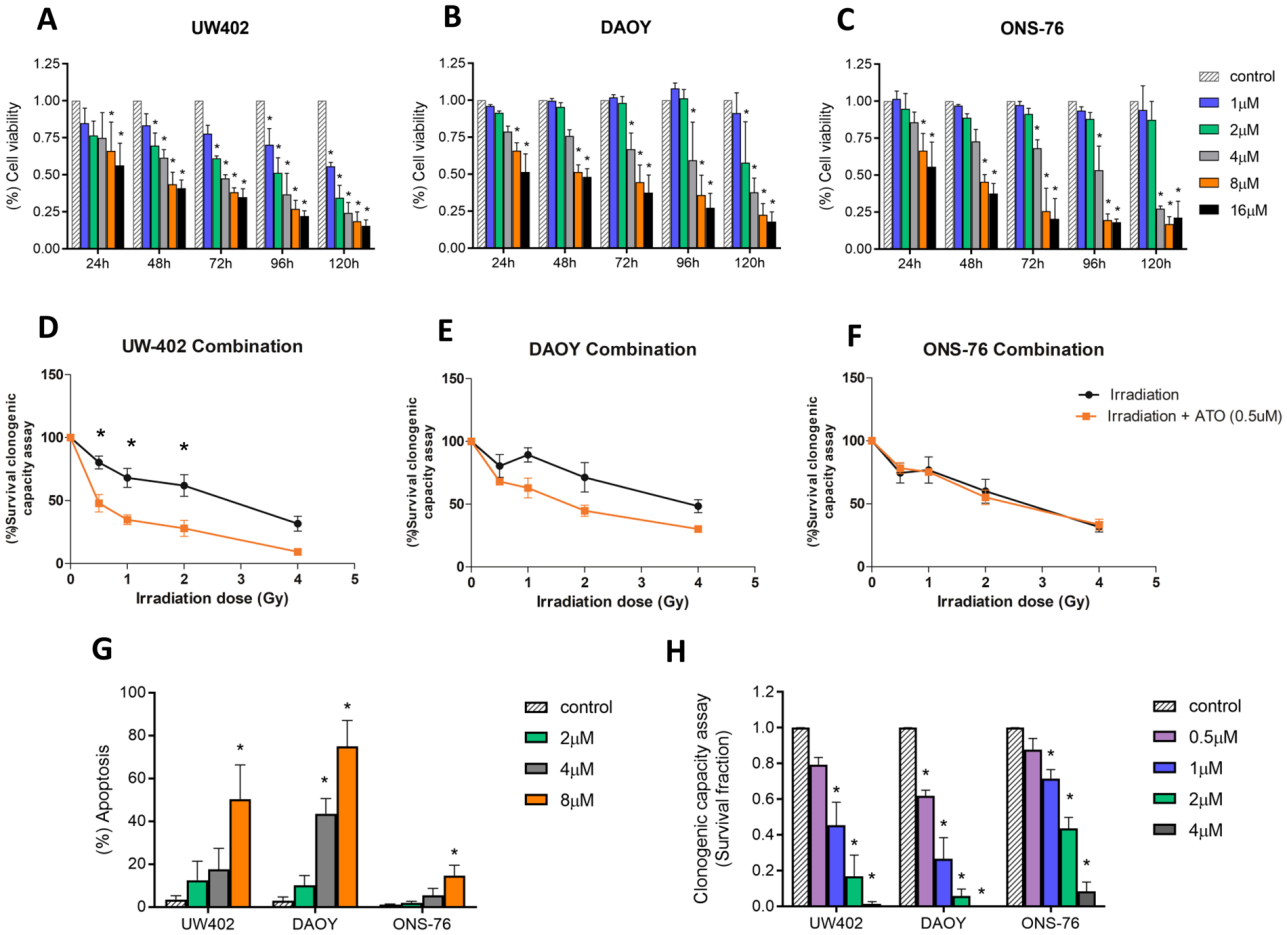
(**A–C**) Cell viability of MB cell lines after treatment with ATO. The assay was carried out for 24, 48, 72, 96 and 120 hours at concentrations of 1, 2, 4, 8 and 16 μM; (**D–F)** ATO radiosensitizing effects in MB cell lines. Cells were treated with ATO 0.5 μM for 48 hours, then they were submitted to radiation at different doses and maintained under standard culture conditions for 7-9 days before colonies analyses; (**G)** Apoptosis rates in UW402, DAOY and ONS-76 cell lines after treatment with ATO (2, 4 or 8 μM) for 48 hours. Cells labeled with annexin and with annexin plus PI were considered; (**H)** Clonogenic capacity assay. Survival fraction of UW402, DAOY and ONS-76 cell lines after treatment with ATO for 48 hours at concentrations of 0.5, 1, 2 and 4 μM. Colonies containing at least 50 cells were considered. Statistical analysis was carried out using one-way ANOVA and Bonferroni post-test. (*) represents p < 0.05. The data reported are representative of three independent experiments.

Treatment with ATO induced a significant reduction of cell viability in a dose-dependent manner for all three cell lines models (Fig. 2A–C), being the UW402 the cell line more affected with lowest IC_50_ values (Table 1). Also, non-neoplastic cells (MRC-5 cell line) were more resistant to ATO effect. While neoplastic cell lines presented a mean reduction of 81.8% in cell viability in the highest dose/time-point, MRC-5 decreased no more than 55.6% (Supplementary Fig. S1). ATO also reduced cell colony formation at concentrations of 0.5, 1, 2 and 4 μM and increased apoptosis rates at 4 and 8 μM after 48 hours of treatment. The clonogenic effects were dose-dependent for all cell lines; however, DAOY showed to be the most sensible model either for apoptosis induction and colony capacity inhibition (Fig. 2G,H). In addition, clonogenic assays combining ATO with irradiation demonstrated that ATO was able to sensitize UW402 cell line (*TP53* mutated) to irradiation, reducing clonogenic capacity from 1.7 to 3.4 times according to doses (0.5, 1, 2 and 4 Gy; p < 0.001), when compared to irradiation alone (Fig. 2D). DAOY (*TP53* mutated) present a marginal radiosensitizing effect, with clonogenic capacity decreasing between 1.2 to 1.6 times (Fig. 2E). Interestingly, ONS-76 cell line (*TP53* wild type) showed none radiosensitizing effect, as observed in Fig. 2F. The Supplementary Table S1 describes the relative clonogenic capacity reductions for all MB cell lines submitted to combined treatment.

**Table 1:** IC50 values for ATO treatments in MB-SHH cell lines.

Cell lines	IC50 values (µM) according to the time of treatment				
	24 h	48 h	72 h	96 h	120 h
UW402	28.8	7.5	4.6	2.4	1.0
DAOY	14.9	10.1	4.6	1.3	3.7
ONS-76	8.1	8.8	6.2	5.0	4.4

In order to investigate the effects of ATO combined with irradiation, we evaluated the expression of key proteins involved in DNA repair of double-strand breaks caused by irradiation. However, the combination of ATO with irradiation did not change the expression of Rad51 (homologous recombination pathway - HR) or Ku86 heterodimer (non-homologous end-joining pathway - NHEJ) (Supplementary Fig. S2). Additional *In silico* analyses were performed with data from a previous study on pediatric MB samples^2^. The results pointed a set of genes involved in the cell cycle, p53 pathways and chromosomal instability that presents specific expression patterns according to the SHH molecular subgroup (Supplementary Table S2). Thus, the expression profile of these genes, together with *TP53* mutation status, showed it to be essential to address ATO’s radiosensitizing effects in pediatric MB-SHH cells.

## Discussion

Currently, the ATO agent is used in the promyelocytic leukemia therapy^11^, but its clinical potential over other types of tumors continue to be underexplored. It has a well-recognized effect in suppressing the SHH pathway by inhibiting GLI proteins, a pathway related to human cancer pathogenesis^7,12,13^. In this study, we observed distinct responses to ATO according to the cell line and *TP53* status. All SHH-MB models had significantly lower cell viability after ATO treatment in a dose-dependent manner. The cells bearing mutations on *TP53* (mainly the DAOY cell line) presented higher sensitivity, showing increased apoptosis rates and reduced cell-colony formation. Previous studies also have shown ATO’s efficiency against HGNET-BCO SHH+/GLI+ tumor resistant to SMO inhibitors (e.g vismodegib)^14,15^. Similar results were observed in other *in vitro* models of pediatrics and CNS tumors (in e.g rhabdomyosarcoma, glioblastoma, leukemia and HGNET-BCOR tumor)^9,10,12,13,16^, but the exact connection between tumor response to ATO and *TP53* mutational status has not been elucidated so far.

The combination of ATO with irradiation therapy decreased colony formation in both *TP53* mutated SHH-MB cell lines, with a significant reduction in UW402 cells. A fair explanation for distinct ATO radiosensitizing effects in pediatric SHH-MB cells rely on molecular differences between the cell lines, which includes *TP53* mutation sites^17,18^. Different point mutations in *TP53* binding sites activate paradoxical cellular programs such as cell death by apoptosis or DNA damage response mechanisms^17,18^. Besides, additional experiments using non-malignant cells are essential to assess whether a combination of ATO with radiation therapy has a selective effect in cancer cells, a study in head and neck carcinoma corroborates with our findings^19^. The authors showed that the combination of ATO with irradiation was shown able to reduce colony formation in p53-deficient cells, which was associated with DNA damage, G2/M arrest, and apoptosis^19^. Moreover, the treatment (ATO + irradiation) was already explored in one cerebellar HGNET-BCO SHH+/GLI+ tumor, that achieved six-months tumor remission with therapy’s proper safety and tolerability^12^. Kumthekar *et al*. (2017)^20^ have confirmed the safety and tolerability of ATO administered at the dose of 0.15 mg/kg/day in combination with focal radiotherapy for infiltrating gliomas of children, which are essential aspects for ATO’s applicability in humans.

Since significant radiosensitizing effects of ATO were found only in one SHH MB *in vitro* model, we hypothesize that alternatives molecular mechanisms are involved in the heterogeneity of SHH MB and thus promote distinct outcomes. We attempted to address this question performing *In silico* analyses using R2 genomic database visualization platform focused on MB SHH subtypes (SHH-alpha, beta, gamma, and delta). Using pathway enrichment analysis on 4 SHH MB subtypes, we found that these entities differ in signatures associated with cell cycle, P53 pathway and chromosomal instability (SHH-alpha, beta, gamma and delta). Similarly, Park and colleagues (2019) described genes from canonical pathways that are prognosis-related and differ according to SHH-MB subtypes (e.g P53 and cell cycle pathways), suggesting that these signatures potentially has an association with tumor aggressiveness and therapy response^21^. Although further analysis are needed, these findings may point towards to a signature set that predicts poor response to therapy within SHH subgroup. Moreover, the novel clinical subtype iSHH (infant patients), a standard to high-risk group, may also benefit from radiosensitizing compounds such as ATO)^14,15^ considering that radiotherapy to treat MB in children below 3 to 5 years is still controversial^15,22^.

Taken together, we showed that ATO induces substantial cytotoxic effect, decreases the clonogenic capacity and induces apoptosis in MB-SHH cells. Interestingly, ATO induced significant radiosensitizing effect over one *TP53* mutated cell line, with no association with Ku86 and Rad51 proteins. The gene expression profiles in MB-SHH samples suggested the cell cycle pathway as a vital prognosis marker, which may be related to the success or failure of combined therapies with irradiation. Therefore, our findings shed new light on a combined therapy of ATO with low-dose irradiation in MB-SHH to be exploited and evaluated by research protocols.

## Material and methods

### Cell culture conditions and reagents

Three SHH-MB cell lines were used in this study: DAOY, UW402 and ONS-76^21^. The SK-ES-1 cell line (EWS-FLI1 oncoprotein) was used as a positive control in cell viability assays and the MRC-5 cell line (Homo sapiens lung Normal fibroblasts - ATCC ® CCL-171™) was used as a non-neoplastic cellular model. DAOY and ONS-76 were cultivated in RPMI medium, while UW402, SK-ES-1 and MRC-5 were cultivated in HAM F10, McCoy and DMEM respectively. All mediums were supplemented with 10% fetal bovine serum, 100 U/mL penicillin and 100 μg/mL streptomycin. Cells were grown under humid atmosphere containing 5% CO_2_ at 37 °C. Cell line authentication was performed by STR DNA Profiling.

The Arsenic Trioxide (ATO) was purchased from Sigma-Aldrich (Product Number: 311383; MO, USA) and was dissolved in alkaline solution of NaOH. After complete solubilization, diluted H2SO4 solution (21.1%) was added for neutralization and the solution was completed with distilled water to a final volume of 250 mL. The stock solution contained 0.1 mg of arsenic trioxide (Solution 0.1 mg L^−1^: 0.25 L).

### DNA extraction and Sanger sequencing

In order to investigate the TP53 mutation status the DNA from DAOY, ONS-76 and UW402 cells was extracted using the Qiamp DNA mini kit according to the manufacturer’s instructions. Amplifications of TP53 Exons (Supplementary Table S3) were performed with 100 ng DNA. PCR products were sequenced by Sanger method using the Big Die™ Terminator Cycle Sequencing Ready Reaction Kit (Applied Biosystems, Courtaboeuf, France) and ABI 3130xL sequencer (Applied Biosystems, Foster City, USA).

### Molecular subgroup assignment of MB cell lines

The total RNA from the three cell lines was isolated with TRIZOL LS reagent (Invitrogen, Carlsbad, CA, USA) and the cDNA was synthesized using the High Capacity kit (Applied Biosystems, Foster City, CA, USA) according to manufacturers’ instructions. The molecular classification was performed by TaqMan Low-Density Array (TLDA PCR-Array). A detailed description of the TDLA method for MB classification can be found elsewhere^23^.

### Cell viability assay

Cell viability was assessed by the Resazurin reduction method. Cells were seeded in 96-well plates and maintained under standard culture conditions for 24 hours (2×10^3^ cells/well). Cells were treated with different ATO concentrations (1 μM to 16 μM) and incubated for 24, 48, 72, 96 or 120 hours. Then, Resazurin solution (Sigma-Aldrich Co., Saint Louis, MO, EUA) was added and the plates were incubated for 4 hours under standard culture conditions. The absorbance at 570 nm wavelength with a reference wavelength of 595 nm was read using an iMax Microplate Reader (Bio-Rad, CA, USA). The IC_50_ values were calculated using Calcusyn software (Biosoft, Ferguson, Missouri, US^24^. Three independent experiments were performed in triplicate.

### Apoptosis assay

Apoptosis was studied by labeling apoptotic cells with Annexin V fluorescein isothiocyanate (BD Biosciences Pharmigen, CA, USA) and necrotic cells with propidium iodide (PI). After ATO treatment for 48 hours (2, 4 or 8 μM), the cells were trypsinized, washed with ice-cold PBS 1X and resuspended in 200 μL of Annexin V binding buffer 1×(BD Biosciences Pharmigen, CA, USA). Cells were labeled with 5 μL of Annexin V and 50 μL of PI solution (50 μM). 10.000 events per treatment were analyzed with a BD FACS Calibur ™ flow cytometer (BD Biosciences Pharmigen, CA, USA). Three independent experiments were performed in triplicate.

### Clonogenicity assay

MB cells were seeded in six-wells plates (500 cells/well), cultivated under standard conditions for 24 hours and treated with ATO (0.5, 1, 2 or 4 μM) for 48 hours. After the treatment period, drug-free medium was added to allow colony growth for approximately 7–9 days. Then, cells were fixed with methanol, stained with 1% Giemsa and analyzed according to Franken *et al*.^25^. For the combination of ATO with irradiation, two experimental groups were considered: cells treated with ATO (0.5 μM) and the cells without treatment. After 48 hours, cells were irradiated with 0, 0.5, 1, 2 or 4 Gy (dose rate = 1.115 Gy/min) using RS-2000 X-Ray apparatus Irradiator Biological System (Rad Source Technologies, Inc., GA, USA). The irradiated cells were maintained under standard culture conditions for 7–9 days before colonies analyses.

### Protein extraction and western blotting

Total protein was extracted using RIPA^®^ lysis buffer (Sigma Aldrich Co., MO, USA), following manufacturer’s recommendations. Equal concentrations (80 μg) of proteins were submitted to 10% SDS-PAGE, followed by transfer to nitrocellulose membrane. The membranes were blocked with 5% (w/v) nonfat milk for one hour and incubated with specific primary antibodies: anti-RAD51 (1:500; overnight; sc-8349; Santa Cruz Biotechnology, TX, USA); anti-Ku86 (1:1000; overnight; sc-1485; Santa Cruz Biotechnology, TX, USA); anti-GAPDH and anti-Vinculin (1:1000; one hour; sc-47724 and sc-25336; Santa Cruz Biotechnology, TX, USA). Finally, the membranes were incubated with species-specific secondary antibodies (1:10.000 anti-rabbit for RAD51; 1:10.000 anti-goat for Ku86; 1:10.000 anti-mouse for GAPDH and vinculin). Chemiluminescent substrate ECL™ (Amersham GE Healthcare, Buckinghamshire, UK) was used and visualized with the ChemiDOC XRS apparatus (Bio-Rad, CA, USA).

### *In silico* analysis in R2 genomic database and visualization tool

Pathway enrichment was performed using R2: Genomics Analysis and Visualization Platform (http://r2.amc.nl) tool. The analysis held a microarray data set from 223 MB-SHH patients in out of 763 MB samples represented by GSE85217. We have focused the analysis on pre-selected SHH subtypes in R2 platform: alpha-beta-gamma-delta. Pathway enriched in each subtype was considered according to the Minimal t-test parameter as p < 0.01 with HugoOnce mode set as “yes”. To visualize and compare specific gene signature from KEGG pathway from: cell cycle, P53 pathway and chromosomal instability among SHH MB subtypes, we have generated a Heatmap using draw Heatmap tool (Z-Score) with pre-set metrics as Pearson correlation distance and average-linkage algorithm.

### Statistical analyses

Data were analyzed by one-way ANOVA followed by the Bonferroni post-test using the Statistical Package for the Social Sciences software (SPSS, Inc., Chicago, USA). Graphs were generated with GraphPad Prism 4.0 (GraphPad Software, San Diego, CA, USA). The level of significance was set at p < 0.05 in all analyses.

### Ethics approval and consent to participate

This research was submitted to and approved by the HC/FMRP- USP Research Ethics Committee and obtained exemption for not involving humans, only commercial cell lines.

## Supplementary information

Dataset 1.
