# Supplementary material for: Arsenic Trioxide exerts cytotoxic and radiosensitizing effects in pediatric Medulloblastoma cell lines of SHH Subgroup
Source: Sci Rep. 2020 Apr 22;10:6836. doi: 10.1038/s41598-020-63808-9 (PMC7176640; doi:10.1038/s41598-020-63808-9)
Supplement: Supplementary file 1 — Dataset 1. [file 41598_2020_63808_MOESM1_ESM.docx]

**Arsenic Trioxide exerts cytotoxic and radiosensitizing effects in pediatric Medulloblastoma cell lines of SHH Subgroup**

Paulo Henrique dos Santos Klinger1;5, Lara Elis Alberici Delsin2, Gustavo Alencastro Veiga Cruzeiro2, Augusto Faria Andrade2, Regia Caroline Peixoto Lira1;6, Pamela Viani de Andrade2, Pablo Ferreira das Chagas2 Rosane Gomes de Paula Queiroz1, Felipe Amstalden Trevisan^3^, Ricardo Santos de Oliveira ^4^, Carlos Alberto Scrideli1, Luiz Gonzaga Tone1,2, Elvis Terci Valera1

^1^Department of Pediatrics; ^2^Department of Genetics; ^3^Division of Radiotherapy; ^4^Division of Pediatric Neurosurgery, Department of Surgeryand Anatomy; Faculty of Medicine of Ribeirão Preto, University of São Paulo, Ribeirão Preto, Brazil.

^5^Instituto de Oncologia Pediátrica IOP/GRAACC, São Paulo, Brazil.

^6^Centro Universitário CESMAC, Maceió-AL, Brazil

To whom correspondence should be sent:

Elvis Terci Valera, MD, PhD

Department of Pediatrics. Ribeirão Preto Medical School, University of São Paulo, São Paulo, Brazil. HC Criança - Av. Bandeirantes, 3900, Ribeirão Preto, SP CEP 14048-900 Brazil.

valeraet@gmail.com

Short title: ATO effects on medulloblastoma SHH group

ORCID IDs

Paulo Henrique dos Santos Klinger: 0000-0001-6739-062X

Augusto Faria Andrade: 0000-0001-5866-0501

Gustavo Alencastro Veiga Cruzeiro: 0000-0002-0005-3984

Regia Caroline Peixoto Lira: 0000-0002-1217-3852

Lara Elis Alberici Delsin: 0000-0002-8452-9141

Pamela Viani de Andrade: 0000-0003-3418-2001

Pablo Ferreira das Chagas: 0000-0002-0652-728X

Rosane Gomes de Paula Queiroz:

Felipe Amstalden Trevisan:

Carlos Alberto Scrideli: 0000-0001-6618-789X

Luiz Gonzaga Tone: 0000-0001-8821-4211

Elvis Terci Valera: 0000-0002-4434-429X

**Supplementary Figure S1.** ATO effect in MRC-5 cell line. **(A)** Cell viability profile of fibroblast cell line MRC-5 treated with 1-16µM doses of ATO for 24h-120h; **(B, C, D, E, F)** Comparison of MRC-5 cell viability profile with neoplastic cell lines DAOY, ONS-64 and UW402 in different time-points, 24h to 120h respectively, according to the doses of ATO treatment. Statistical analysis was done using two-way ANOVA and bonferroni`s multiple comparisons test. The (*) represents p<0.05.

**Supplementary Figure S2.** ATO did not reduce the expression of the Rad51 (37 kDa) and Ku86 (86 kDa) proteins which are important for the repair of double strand breaks caused by irradiation through the homologous (HR) recombination pathway. DAOY **(A-C)** and ONS-76 **(B-D)** under conditions: **(1)** Cell line without treatments (Control); **(2)** ATO treatment (0.5 uM) for 48 hours; **(3)** Irradiation (0.5 Gy); **(4)** Combined treatment: 0.5 uM of ATO for 48 hours followed by irradiation (0.5 Gy). For protein loaded in lanes 3 and 4, the cells pellets were collected four hours after irradiation.

**Supplementary Figure 1**

**Supplementary Figure 2**


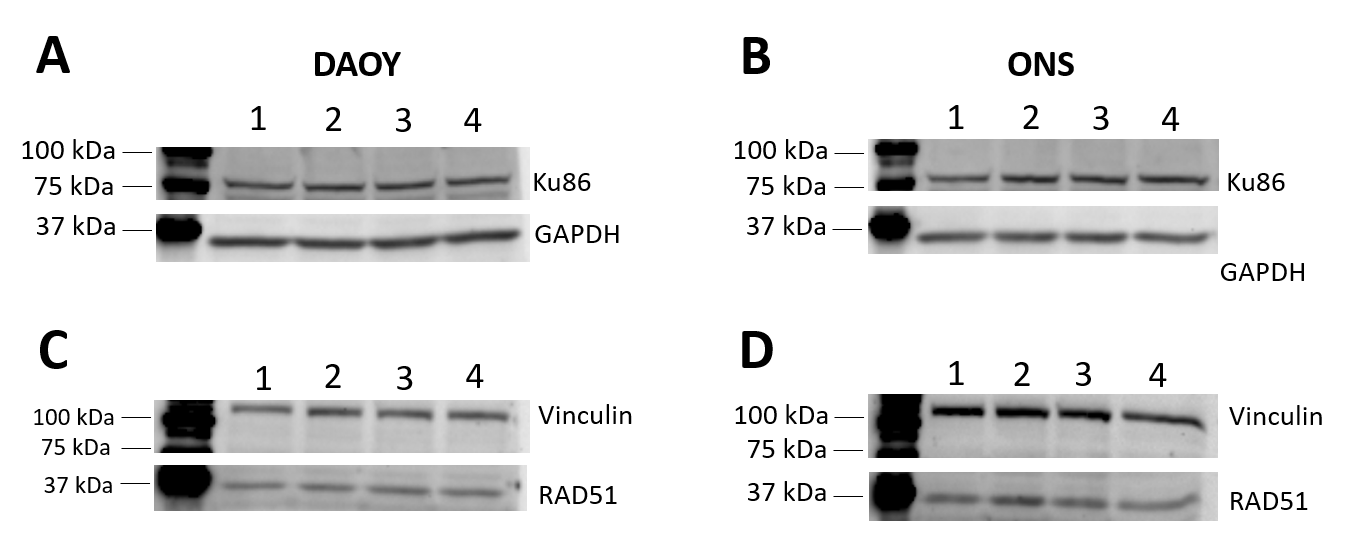


**Supplementary Tables**

**Supplementary Table S1.** Clonogenic capacity reduction in MB cell lines. Irradiation alone relative to the combination ATO+irradiation

| **Cell lines** | **Relative values:**  **Irradiation / ATO+irradiation** | | | | |
| --- | --- | --- | --- | --- | --- |
|  | **0 Gy** | **0.5 Gy** | **1 Gy** | **2 Gy** | **4 Gy** |
| **UW402** | 1 | 1.7 | **2.0** | **2.2** | **3.4** |
| **DAOY** | 1 | 1.2 | 1.4 | 1.6 | 1.6 |
| **ONS-76** | 1 | 1 | 1 | 1.1 | 0.9 |

# Colors are proportional to clonogenic capacity reduction: No reduction, white; Slight reduction, light gray; Moderate reduction, dark grey, and Intense reduction, black.

**Supplementary Table S2.** List of genes found by *in silico* analyses that are involved in cell cycle, p53 pathways and chromosomal instability.

| **GENES:** | *EI24*  *CYCS*  *CCNE1*  *PMAIP1*  *CCND2*  *CDK4*  *CHECK1*  *CDK2*  *CCNE2*  *CHECK2*  *CDK1*  *RRM2*  *GTSE1*  *CCNB2*  *CCNB1* |
| --- | --- |

**Supplementary Table S3.** Primers sequences used for Sanger Sequencing of *TP53* gene.

| **Exons** | **Base pairs (bp)** | **Sense sequence** | **Antisense sequence** |
| --- | --- | --- | --- |
| 2 + 3 | 470 | 5’ CTGTCTCAGACACTGGCATGG 3’ | 5’ GGCAAGGGGGACTGTA 3’ |
| 4 | 591 | 5’ GGACTGACTTTCTGCTCTTGTCT 3’ | 5’ CAGAGATCACACATTAAGTGGGT 3’ |
| 5 | 472 | 5’ CTCTCTAGCTCGCTAGTGGGT 3’ | 5’ CGAAAAGTGTTTCTGTCATCCAA 3’ |
| 6 | 396 | 5’ GCCATGGCCATCTACAAGCA 3’ | 5’ TGGGGTTATAGGGAGGTCAAA 3’ |
| 7 | 325 | 5’ ACAGGTCTCCCCAAGG 3’ | 5’ AAACTGAGTGGGAGCAGTAAGGA 3’ |
| 8 + 9 | 499 | 5’ GGACAAGGGTGGTTGGGAGTAGA 3’ | 5’ CCCAATTGCAGGTAAAACAGTCA 3’ |
| 10 | 421 | 5’ CAGTTTCTACTAAATGCATGTTG 3’ | 5’ ATACACTGAGGCAAGAATGTGGT 3’ |
| 11 | 380 | 5’ CATCTTGATTTGAATTCCCGTTG 3’ | 5’ CACCAGTGCAGGCCAACTTGTTC 3’ |
